# Supplementary material for: Giant Fern Genomes Show Complex Evolution Patterns: A Comparative Analysis in Two Species of Tmesipteris (Psilotaceae)
Source: Int J Mol Sci. 2023 Jan 31;24(3):2708. doi: 10.3390/ijms24032708 (PMC9916801; doi:10.3390/ijms24032708)

Figure S1. Graph generated with RepeatExplorer2 comparing the proportion of reads contained in each cluster for *Tmesipteris obliqua* and *T. tannensis*.

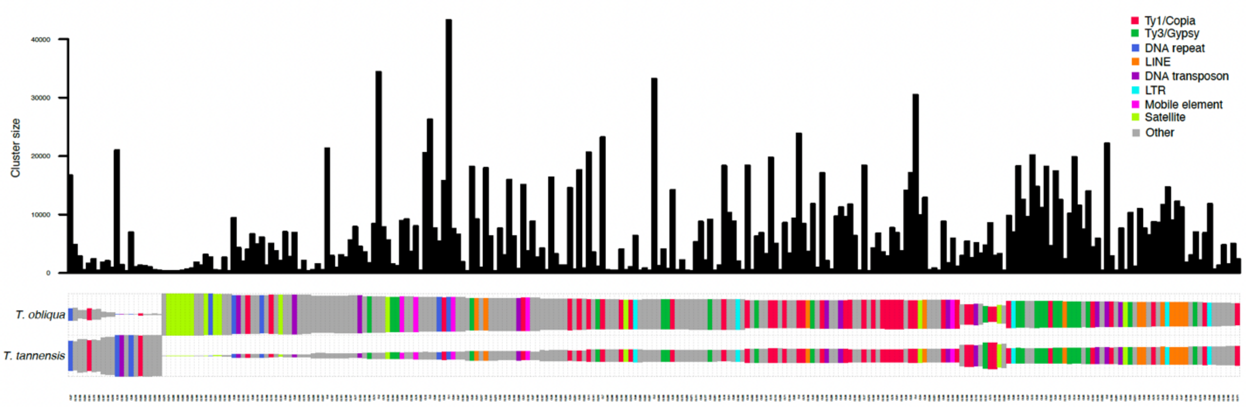

Figure S2. Intrafamily heterogeneity of repeats in *Tmesipteris*. (A-F) Histograms of percentage sequence similarity for read pairs from selected repeat families from *T. tannensis* and *T. obliqua*. The y axis “count” refers to the number of reads and dashed lines indicate the median values of heterogeneity per cluster.

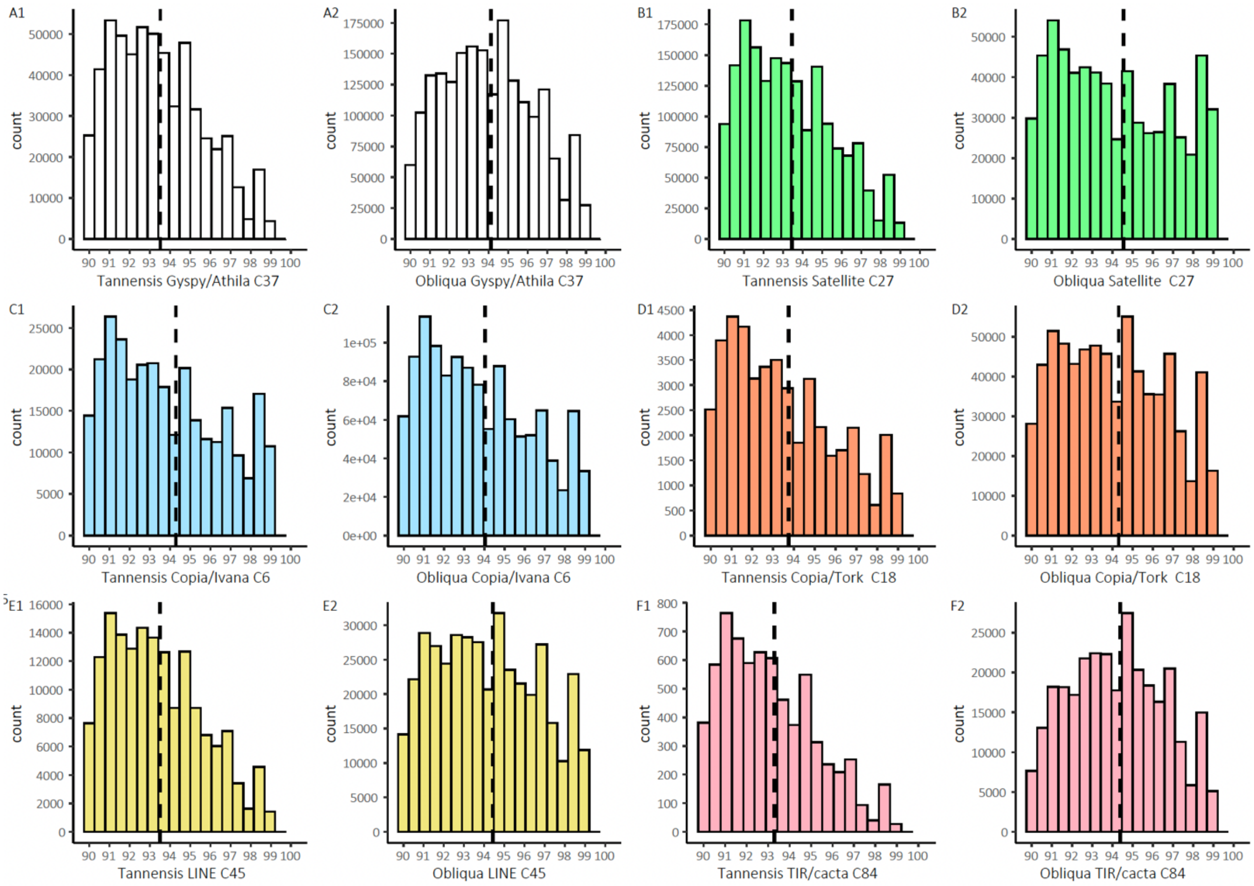

Supplement: Supplementary file 1 [file ijms-24-02708-s001.zip › Figures S1 and S2.pdf]
