# Supplementary material for: Giant Fern Genomes Show Complex Evolution Patterns: A Comparative Analysis in Two Species of Tmesipteris (Psilotaceae)
Source: Int J Mol Sci. 2023 Jan 31;24(3):2708. doi: 10.3390/ijms24032708 (PMC9916801; doi:10.3390/ijms24032708)
Supplement: Supplementary file 1 [file ijms-24-02708-s001.zip › Table S1.pdf]

Table S1. Details per individual of genome skimming and individual clustering analysis

Individual code	GS 1C (Gbp)*	Chrom. number (2n)**	Sequencing run number	Individual clustering	
				No. reads	Coverage (× 1C)
T.obliqua	147.29	416	SRR22253889	4,000,000	0.0027 ×
T.tannensis	73.19	208	SRR22253888	4,000,000	0.0055 ×

*Nuclear DNA contents and **Chromosome number from:

T. tannensis:

*/**Clark, J.; Hidalgo, O.; Pellicer, J.; Liu, H.; Marquardt, J.; Robert, Y.; Christenhusz, M.; Zhang, S.; Gibby, M.; Leitch, I.J.; et al. Genome Evolution of Ferns: Evidence for Relative Stasis of Genome Size across the Fern Phylogeny. *New Phytol.* **2016**, *210*, 1072–1082, doi:10.1111/nph.13833

T.obliqua:

*Hidalgo, O.; Pellicer, J.; Christenhusz, M.J.M.; Schneider, H.; Leitch, I.J. Genomic Gigantism in the Whisk-Fern Family (Psilotaceae): *Tmesipteris Obliqua* Challenges Record Holder *Paris Japonica*. *Bot. J. Linn. Soc.* **2017**, *183*, 509–514, doi:10.1093/botlinnean/box003.

Tindale, M.D.; Roy, S.K . A cytotaxonomic survey of the Pteridophyta of Australia. *Australian Syst. Bot.* **2002, 15, 839–937, doi.org/10.1071/SB00034
